# Changes in ocular morphology after cataract surgery in open angle glaucoma patients

**DOI:** 10.1038/s41598-021-91740-z

**Published:** 2021-06-09

**Authors:** Giedre Pakuliene, Loreta Kuzmiene, Brent Siesky, Alon Harris, Ingrida Januleviciene

**Affiliations:** 1 Department of Ophthalmology, Lithuanian University of Health Sciences, Eiveniu g. 2, 50161 Kaunas, Lithuania; 2 Icahn School of Medicine at Mount Sinai, New York, NY USA

**Keywords:** Anatomy, Lens diseases, Ocular hypertension

## Abstract

The purpose of this study was to evaluate intraocular pressure (IOP) pre- and postoperatively, together with anterior chamber angle (ACA) parameters and biometrical results in cataract patients with or without open angle glaucoma (OAG). The prospective observational case–control study included 15 eyes with cataract and OAG in the glaucoma group and 25 eyes with only cataract in control group. Examination included full ophthalmic evaluation, IOP, ocular biometry and anterior segment optical coherence tomography measuring ACA pre- and 6 months postoperatively. OAG patients had a larger absolute IOP reduction compared to control group. Anterior chamber depth (ACD) and ACA width significantly increased in both groups. The OAG group had a tendency of narrower ACA preoperatively, but overall ACA parameters did not differ in either group pre- and postoperatively. The ACD change after surgery correlated with ACA parameters in the control group, but not in OAG group. Axial length was shorter postoperatively in the control group, but remained similar in the OAG group. Absolute IOP reduction was more pronounced in cataract patients with OAG than in cataract patients without glaucoma. ACD and ACA postoperatively increased in both groups and AL shortening was observed in non-OAG in cataract group.

## Introduction

The conditions of anterior chamber angle (ACA) structures highly impact aqueous humour drainage and intraocular pressure (IOP)^1–4^. Over time the ACA tends to decrease with age, as lens thickness increases^5^. Cataract has been shown to enhance these changes^6^ while the surgical removal of cataracts may alter IOP and produce other physiological and morphological changes within ocular tissues.

Elevated IOP is a significant risk factor for ocular pathologies such as glaucoma. Despite its importance, the results concerning IOP reduction after cataract surgery are heterogeneous and not uniformly presented in the different studies available in the literature^4, 7–21^. When examining the data, open angle glaucoma (OAG) patients, who have higher IOP prior to cataract surgery, tend to have a more significant IOP reduction following cataract surgery^16^ while ACA parameters in OAG patients have been shown to have similar characteristics as non-OAG patients^22^. Despite being a significant risk factor for OAG progression, IOP changes post cataract surgery remain poorly understood. The mechanism(s) behind IOP reduction post cataract removal have several interconnected theories: (1) the anterior chamber anatomy changes, as the intraocular lens (IOL) is significantly thinner than the crystalline lens; (2) the anterior chamber deepens, the width of anterior chamber increases enabling higher volume of aqueous humour to reach trabecular meshwork; and (3) the resistance in trabecular meshwork also decreases and the ciliary body and its’ processes change position leading to decreased IOP^2^.

Importantly, most of the studies concerning ACA and IOP change after cataract surgery investigated closed angle glaucoma^23–25^. The main mechanisms related to these changes were ACA opening and increased aqueous drainage through the trabecular meshwork^2^. The reduction of IOP was also observed in eyes with OAG^11–21^. However information on the ACA has received significantly less attention in the literature even though changes in ACA might still be a major influencing risk factor. Specifically, the studies investigating ACA changes in OAG patients typically do not have control groups without glaucoma, and/or were compared with angle closure glaucoma^13, 14, 18, 21^. Another interesting element missing in the current literature is an understanding of IOP change after phacoemulsification and the ability to account for ocular biometrical dynamics in cataract patients with or without OAG. For example, some studies indicate AL shortening after cataract surgery^10, 26^, while others do not find statistical significance^27, 28^. Despite their potential importance, these important considerations are not currently widely considered when evaluating post-operative IOP.

The combined relevance of these factors and OAG risk are high, however the literature does not readily have data from published studies investigating all of these interconnected aspects of IOP in cataract patients. Therefore, the purpose of this study was to concurrently evaluate ACA parameters change after cataract surgery, in perspective of IOP and ocular biometry results in cataract patients with or without OAG before and after phacoemulsification and IOL implantation to fill in the missing associations between these important physiological biomarkers of risk.

## Results

Fifteen eyes with cataract and OAG and 25 eyes with only cataract that met inclusion criteria were included into the study. Patients’ age and ethnicity were not significantly different between the groups (Table 1).

**Table 1:** Demographic data.

	Control	OAG	*p*
Number of eyes, n	25	15	–
Gender female, n (%)	16 (64%)	12 (80%)	–
Age mean (SD), years	74.25 (6.3)	74.46 (9.3)	> 0.05 (Mann–Whitney U test)
Ethnicity Caucasian (%)	100%	100%	**–**

The age difference between groups was insignificant, there were more female than male patients in both groups.

All of the patients were Caucasian.

Preoperative IOP was statistically significantly higher in OAG group than in control group. Statistically significant IOP reduction was observed 6 months after the surgery in both groups (*p* < 0.05, Wilcoxon Signed Rank Test) (Table 2). Postoperative IOP was lower in the OAG group than in the control group, but the difference was not statistically significant (Table 2). The change in IOP mean (SD) was − 1.8 (2.7) mmHg in control group and (SD) − 4.7 (2.8) mmHg in OAG group (*p* < 0.001, Mann–Whitney U Test). All of the OAG group patients received the same medical anti-glaucomatous treatment before and after cataract surgery.

**Table 2:** IOP pre- and postoperatively (Significance in bold).

	Control	OAG	*p*
IOP mean (SD), mmHg preop	14.9 (2.7)	17.1 (2.5)	**0.023 (Mann–Whitney U Test)**
IOP mean (SD), mmHg 6 months postop	13.1 (2.2)	12.5 (3.2)	0.367 (Mann–Whitney U Test)
*p*	**0.001 (Wilcoxon Signed Rank Test)**	**0.004 (Wilcoxon Signed Rank Test)**	

The preoperative IOP was higher in OAG group. The postoperative IOP was similar between the groups. IOP decreased significantly in both groups after cataract surgery.

Preoperatively, there were no statistically significant differences in AL, ACD and lens thickness between control and OAG groups (Table 3).

**Table 3:** Biometry results pre- and postoperatively (Significance in bold).

	Control	OAG	*p*
**AL mean (SD), mm**			
Preoperatively	23.23 (1.0)	22.84 (1.1)	0.267 (Mann–Whitney U Test)
Postoperatively	23.07 (1.0)	22.91 (1.2)	0.847 (Mann–Whitney U Test)
*p*	**< 0.001 (Wilcoxon Signed Rank Test)**	0.257 (Wilcoxon Signed Rank Test)	
**ACD mean (SD), mm**			
Preoperatively	3.02 (0.3)	2.9 (0.3)	0.148 (Mann–Whitney U test)
Postoperatively	4.6 (0.5)	4.5 (0.4)	0.658 (Mann–Whitney U test)
*p*	**< 0.001 (Wilcoxon Signed Rank Test)**	**0.001 (Wilcoxon Signed Rank Test)**	
**LT mean (SD), mm**	4.03 (0.3)	4.15 (0.3)	0.215 (Mann–Whitney U test)
LT min, mm	4.03	4.15	
LT max, mm	5.21	5.24	
**IOL thickness mean (SD), mm**	0.93 (0.3)	0.79 (0.2)	0.091 (Mann–Whitney U test)
**CCT mean (SD), μm**			
Preoperatively	568.8 (41.2)	530.9 (33.2)	**0.003 (Mann–Whitney U test)**
Postoperatively	561.8 (46.5)	531.1 (33.2)	**0.046 (Mann–Whitney U test)**
*p*	0.249 (Wilcoxon Signed Rank Test)	0.669 (Wilcoxon Signed Rank Test)	–

*AL* axial length (mm) pre- and postoperatively, *ACD* anterior chamber depth (mm) pre-and postoperatively, *LT (mm)* lens thickness mean, as well as minimum and maximum values in each group, *IOL thickness (mm)* intraocular lens thickness, *CCT (mm)* central corneal thickness (μm) pre- and postoperatively.

After phacoemulsification and IOL implantation, AL shortening of ≥ 0.1 mm of was observed in 92% cases (n = 23) in control group, while in OAG group the shortening of AL was observed in 26.6% of cases (n = 4). IOLMaster 700’ repeatability SD was considered 0.008 mm^29^. The statistically significant AL decrease 6 months postoperatively was observed in the control group. We observed a tendency to increase in AL in OAG group, but the difference was not statistically significant (Table 3).

Mean ACD statistically significantly increased in both groups after the surgery, but the difference between the groups was not significant (Table 3). In control group, the ACD change range was 1.06 to 2.62 mm and in OAG group, the ACD change range was 0.83 to 2.75 mm, The change in ACD did not correlate with IOP reduction in both groups (*p* > 0.05, Spearman’s correlation coefficient).

CCT was thinner in OAG group than in control group pre- and postoperatively. Lens thickness and IOL thickness were not statistically significantly different in both groups. The mild IOL difference between groups could be associated with the refractive status. (Table 3).

**Figure 1:**
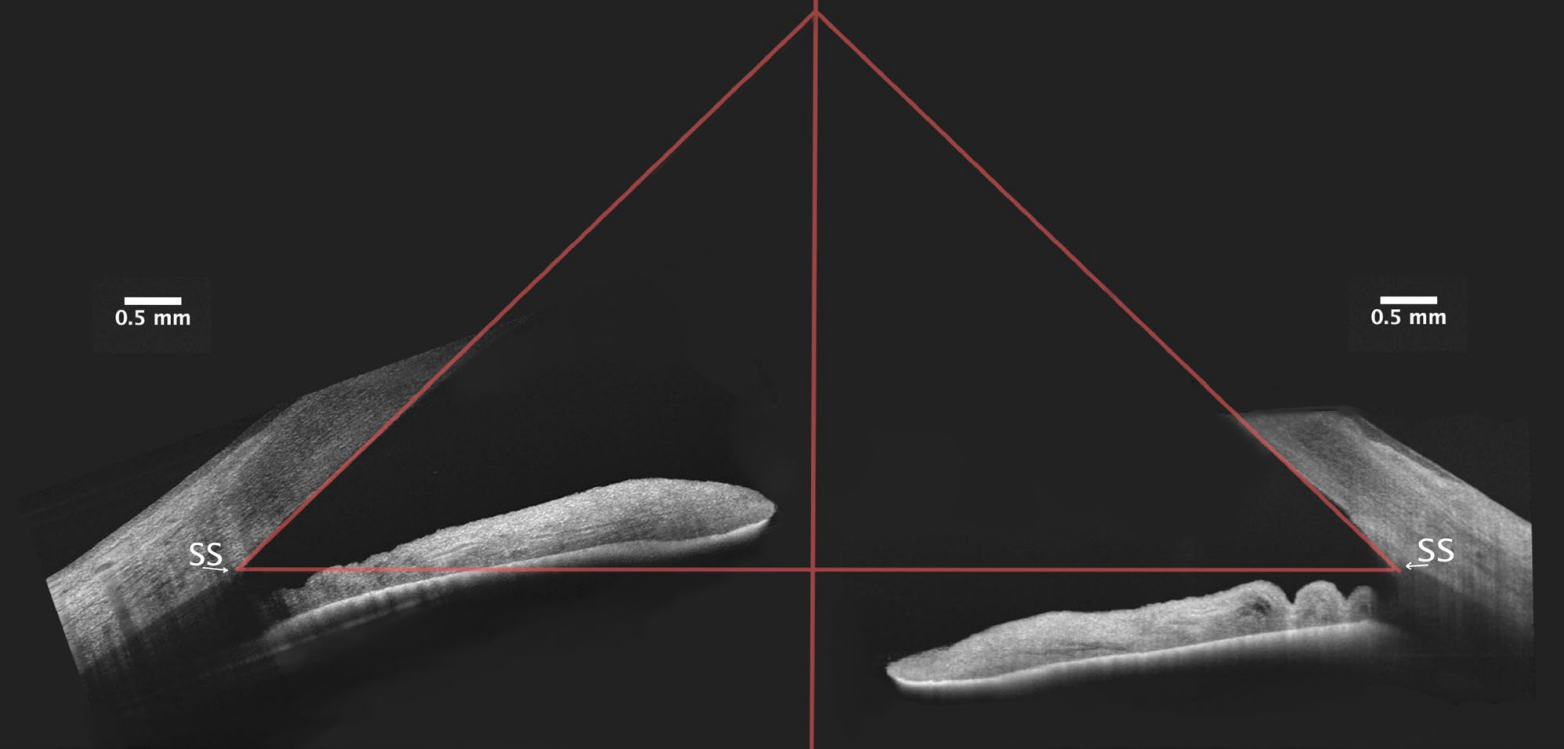
ACA before (left) and 6 months after (right) phacoemulsification and IOL implantation. The orientation of tomograms was adjusted for demonstrational purposes. Both of the sides represented temporal tomogram of the right eye pre- (left) and postoperatively (right). The lining point in both tomograms was the sclera spur (SS). Preoperatively, iris had more upward orientation than postoperatively. The ACA was open pre- and postoperatively. (Images acquired with DRI OCT Triton plus (Ver.10.13).

Preoperative AOD500, TISA500 and TISA 750 were similar in control and OAG groups (*p* > 0.05, Mann–Whitney U Test). ACA parameters increased (AOD500, AOD750, TISA500) postoperatively in both groups (*p* > 0.05, Wilcoxon Signed Ranks Test) (Fig. 1). ACA depth increased significantly after the surgery in both groups, but postoperative difference between the groups was insignificant (Table 4).

**Table 4:** ACA parameters pre- and postoperatively (Significance in bold).

	Control	OAG	*p*
**Nasal AOD500 (mm)**			
Preoperatively	0.41 (0.20)	0.34 (0.11)	> 0.05 (Mann–Whitney U Test)
Postoperatively	0.64 (0.17)	0.64 (0.20)	> 0.05 (Mann–Whitney U Test)
*p*	**< 0.001 (Wilcoxon Signed Ranks Test)**	**0.003 (Wilcoxon Signed Ranks Test)**	–
**Temporal AOD500 (mm)**			
Preoperatively	0.42 (0.16)	0.38 (0.16)	> 0.05 (Mann–Whitney U Test)
Postoperatively	0.66 (0.18)	0.70 (0.23)	> 0.05 (Mann–Whitney U Test)
*p*	**< 0.001 (Wilcoxon Signed Ranks Test)**	**< 0.001 (Wilcoxon Signed Ranks Test)**	–
**Nasal AOD750 (mm)**			
Preoperatively	0.56 (0.25)	0.54 (0.20)	> 0.05 (Mann–Whitney U Test)
Postoperatively	0.85 (0.25)	0.85 (0.23)	> 0.05 (Mann–Whitney U Test)
*p*	**< 0.001 (Wilcoxon Signed Ranks Test)**	**0.004 (Wilcoxon Signed Ranks Test)**	–
**Temporal AOD750 (mm)**			
Preoperatively	0.55 (0.24)	0.47 (0.14)	> 0.05 (Mann–Whitney U Test)
Postoperatively	0.94 (0.24)	0.99 (0.30)	> 0.05 (Mann–Whitney U Test)
*p*	**< 0.001 (Wilcoxon Signed Ranks Test)**	**0.001 (Wilcoxon Signed Ranks Test)**	–
**Nasal TISA500 (mm)**			
preoperatively	0.16 (0.07)	0.14 (0.05)	> 0.05 (Mann–Whitney U Test)
postoperatively	0.23 (0.06)	0.23 (0.07)	> 0.05 (Mann–Whitney U Test)
*p*	**< 0.001 (Wilcoxon Signed Ranks Test)**	**0.002 (Wilcoxon Signed Ranks Test)**	–
**Temporal TISA500 (mm)**			
Preoperatively	0.16 (0.06)	0.15 (0.08)	> 0.05 (Mann–Whitney U Test)
Postoperatively	0.27 (0.16)	0.23 (0.08)	> 0.05 (Mann–Whitney U Test)
*p*	**< 0.001 (Wilcoxon Signed Ranks Test)**	**0.008 (Wilcoxon Signed Ranks Test)**	–

AOD500, angle opening distance at 500 μm from scleral spur; AOD750, angle opening distance at 750 μm from scleral spur; TISA500, trabecular-iris space area 500 μm from scleral spur. All of the measurements are presented at nasal and temporal quadrants pre- and postoperatively.

In control group, the postoperative AOD500 change range nasally was 0.06 to 0.64 mm, the mean (SD) change was 0.23 (0.15) mm; temporally was 0.02 to 0.68 mm, the mean (SD) change was 0.24 (0.15) mm. In OAG group, the postoperative AOD500 change range nasally was 0.08 to 0.64 mm, the mean (SD) change was 0.31 (0.19) mm; temporally was 0.02 to 0.77 mm, the mean (SD) change was 0.34 (0.23) mm.

**Figure 2:**
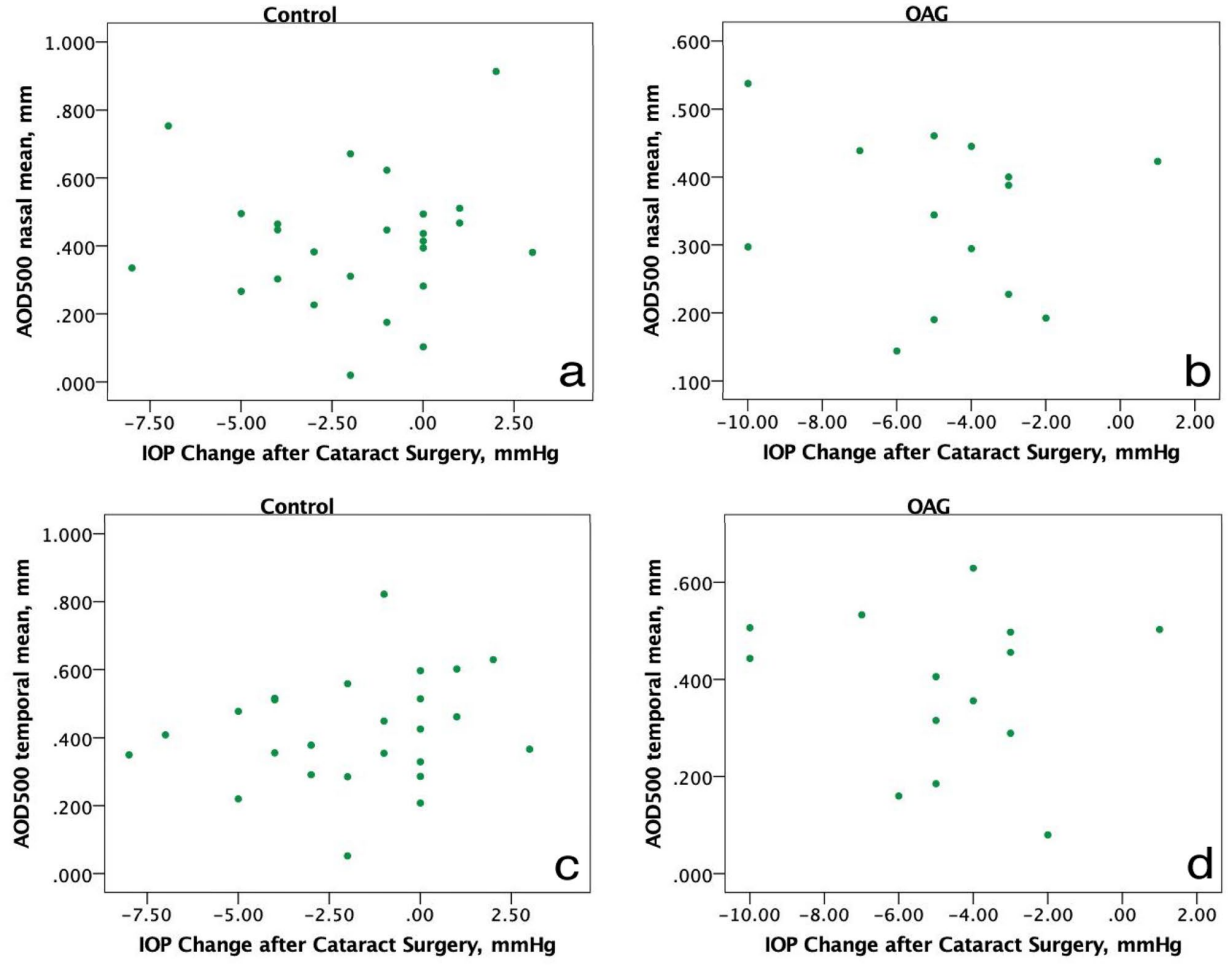
Correlations between IOP and preoperative AOD500 nasally (**a**,**b**) and temporally (**c**,**d**). IOP change postoperatively did not correlate with AOD500 in both groups. (Illustration made using IBM SPSS Statistics for Windows, Version 23.0).

The IOP change range was − 8.0 to + 3.0 mmHg in control group and − 10.0 mmHg to + 1.0 mmHg in OAG group. We found, that IOP change 6 months postoperatively did not correlate with preoperative AOD500 nasally and temporally in both groups (Spearman’s ρ > 0.05) (Fig. 2).

**Figure 3:**
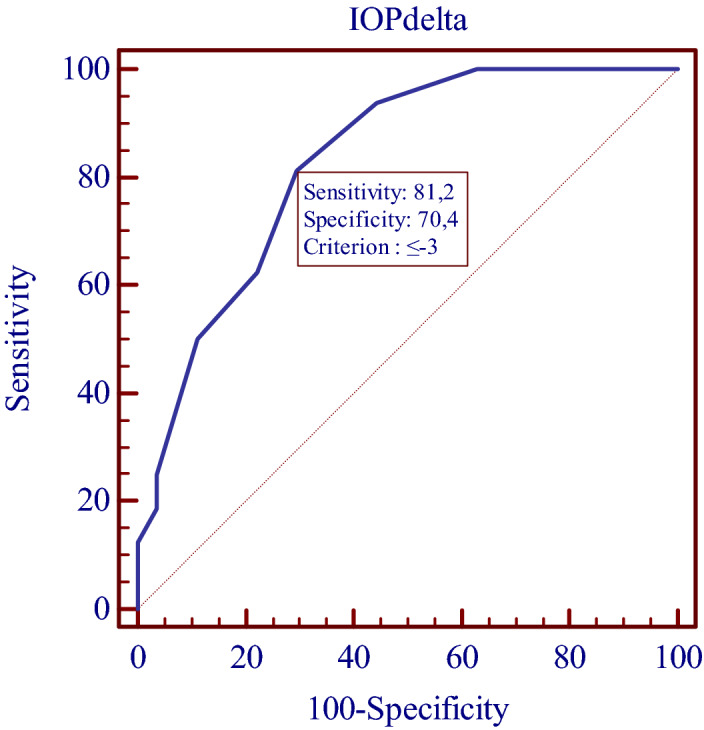
ROC analysis. IOP cut-off value was < − 3.0 mmHg. Sensitivity 81.2%, Specificity 70.4% Area under ROC curve 82.9%. (Illustration made using IBM SPSS Statistics for Windows, Version 23.0).

The ROC analysis was performed for IOP change in both groups. The IOP cut-off value was found to be < − 3.0 mmHg. Sensitivity 81.2%, Specificity 70.4%. Area under ROC curve 82.9%. IOP value of < − 3.0 mmHg was observed in control group (n = 8, 29.6%), and in OAG group (n = 13, 81.3%) (p = 0.001). If the patient had OAG, IOP change was ≤  − 3.0 mmHg (Odds ratio 10.292 (CI 2.29–46.252) (Fig. 3).

We found moderate positive correlation in the control group between postoperative ACD change and AOD500 change nasally (*p* = 0.011, Spearman’s ρ = 0.513) and temporally (*p* = 0.009, Spearman’s ρ = 0.501). We did not observe similar connection in OAG group nasally (*p* = 0.102, Spearman’s ρ = 0.455) and temporally (*p* = 0.970, Spearman’s ρ = 0.011).

## Discussion

In the present study, we compared IOP change in OAG patients with cataract and controls (cataract without glaucoma) finding that OAG patients had a larger absolute IOP reduction (mmHg) compared to control group (Table 2). Importantly, while the postoperative IOP in our study was similar in both groups the medically controlled OAG patients had significantly higher mean preoperative IOP than the control group, which may have influenced the results. As for ROC analysis result, it was found that, if the patients had OAG, they were more likely to have IOP change of ≤ − 3.0 mmHg, than if they did not have OAG.

The results of IOP change after cataract surgery varied between different authors (Table 5). Factors identifying patients, who could expect larger IOP drop, are still under review. Majstruk et al. found that IOP varied from − 5 to + 5 mmHg in POAG patients 1 year after phacoemulsification and IOL implantation^15^. In our study we found agreement, as some patients also did not have IOP reduction after phacoemulsification and IOL implantation. Preoperative and postoperative ACA biometry parameters could be factors in determining changes or lack thereof in IOP.

**Table 5:** Studies, evaluating ΔIOP in OAG and non-glaucomatous cataract patients, conducted in last 10 years, measured with Goldmann applanation tonometry.

	Diagnosis	ΔIOP, mmHg	Time of final measurement	Significance, *p*
DeVience et al.^7^	No glaucoma	− 1.3 (2.6)	3 years	–
Huang et al.^4^	No glaucoma	− 1.99 (2.7)	3 months	< 0.05
Beato et al.^8^	No glaucoma	− 2.4 (2.8)	6 months	< 0.05
Lv et al.^9^	No glaucoma			
	Emmetropia	﻿− 2.9 (2.9)	90 days	< 0.05
	Mild-moderate myopia	﻿− 3.6 (2.9)		< 0.05
	High myopia	− 2.7 (1.5)		< 0.05
Bilak et al.^10^	No glaucoma	− 2.45 (3.78)	1 month	< 0.05
Moghimi et al.^30^	No glaucoma	− 4.95 (2.26)	3 months	< 0.05
Baek^11^	No glaucoma OAG	− 1.03 (3.72) − 1.08 (3.79)	1 year	< 0.05 < 0.05
Coh et al.^12^	No Glaucoma OAG	− 2.8 (3.83) − 2.66(2.07)	4 months	< 0.05 < 0.05
Kim et al.^13^	OAG	No change	1–16 months	> 0.05
Kim et al., 2016 ^14^	OAG	− 0.87 (2.43)	6 months	> 0.05
Majstruk et al.^15^	OAG	− 1.15 (3)	1 year	< 0.05
Guan et al.^16^	OAG	− 1.8 (3.5)	1 year	< 0.05
Iancu^17^	OAG, uncontrolled	− 1.9 (3.9)	1 year	< 0.05
Siak et al.^18^	OAG	− 2.1	1 year	> 0.05
Yoo et al.^19^	OAG	− 2.2 (2.5)	3 months	< 0.05
Jimenez-Roman et al.^20^	OAG	− 2.8	1 year	< 0.05
Lin et al. ^21^	OAG			
	Narrow angle	− 4.2 (3.0)	1 year	< 0.05
	Wide angle	− 2.2 (3.0)		< 0.05

Studies, evaluating ΔIOP in OAG and non-glaucomatous cataract patients, conducted in last 10 years, measured via Goldmann applanation tonometry.

We chose to evaluate ACA parameters before and 6 months after cataract surgery, because different studies suggest IOP change evens out over period of time^31, 32^. Our data suggests that preoperative ACA width does not correlate with IOP change 6 months postoperatively. The ACA width increased in both groups postoperatively. The increase of ACA postoperatively did not correlate with reduction of IOP in both groups as well. According to Lin et al., ACA parameters preoperatively had an impact on postoperative IOP in glaucoma patients (the patients were graded according to angle appearance wide to narrow angle, where narrow angle predetermined larger IOP reduction 1 month postoperatively)^21^. In our study, the patients, who had the highest IOP reduction in both groups did not have AOD500 below 200 μm^33^, which means they did not have narrow ACA (Fig. 2). This suggests another mechanism alongside the ACA opening after phacoemulsification and IOL implantation. One such factor could be trabecular meshwork remodeling and ciliary body fibrosis after cataract surgery^34^.

As presented by Shammas et al.^6^, cataract mostly affects thickening of anterior cortex space of the lens. This could push iris anteriorly and reduce depths of ACA. In our study, ACA parameters were similar among control and OAG groups, while the reduction of IOP differed significantly. OAG showed the tendency of narrower ACA parameters, but the difference was not significant. Lens thickness among both groups was also similar, without marginal extreme values (Table 3). This suggests that deepening of ACA is not the single factor related to decrease of IOP after cataract surgery.

The AOD500, TISA500 and TISA750 after phacoemulsification and IOL implantation increased significantly in both groups. Our findings were similar to the results acquired by Kim et al.^13^, where they studied ACA changes in cataract patients without OAG, however, the authors did not include IOP in their study.

Lee et al. found, that deepening of ACD after phacoemulsification did not correlate with changes in ACA parameters in non-glaucomatous patients. We found, that there was a moderate positive association in non-glaucomatous cataract patients between ACD change and ACA change nasally and temporally. The same association was not found in OAG patients^35^. ACD and ACA relationship can be partly explained by the natural lens and IOL position in the eye. The natural lens before cataract surgery touches the posterior surface of the iris. When we perform cataract surgery and implant an IOL, the IOL does not touch the posterior surface of the iris^36^. Therefore, the ACD and ACA change after cataract surgery differ. In agreement, we also found, that ACA change after cataract surgery was highly variable in both groups.

Anterior segment optical cogherence tomography (AS-OCT) ACA assessment are not in complete agreement with gonioscopy (the gold standard of ACA assessment), as AS-OCT shows more false positive narrow or closed ACA^37, 38^. However, AS-OCT allows very precise ACA measurement and can be used for evaluating anterior chamber dynamics after certain procedures. The conditions, under which the measurements are completed, are of key importance^39^.

Along with IOP, ocular structure may have profound effects on OAG risk. In our study, we found that AL was significantly shorter after cataract surgery in patients in control group, but remained similar in patients with cataract and OAG. AL shortening after cataract surgery was debated in different studies (Table 6) with no consensus in the literature. The absolute change in AL length after cataract surgery and significance differed among the studies. All of the found studies were with short follow up and did not evaluate glaucoma patients (Table 6)^10, 26–28^. Specifically, Huang et al. found in their study, that axial length decreases with age^40^ which may affect long term results. Comparatively, the follow up period of time in our study was longer which provided some insight into what may be expected during long-term evaluation post cataract removal. It is also important to note that Bernardo et al. suggested that pseudophakic eyes required “aphakic” option in IOLMaster 500 biometry to reduce AL measurement error^28^. In our study we used the “pseudophakic” option and IOLMaster 700 for our measurements.

**Table 6:** AL difference before and after phacoemulsification and IOL implantation. All of the patients, described in the literature, did not have glaucoma.

	Diagnosis	ΔAL	Period of time	Mean AL preop	Mean AL postop	p
Bilak et al.^10^	No glaucoma	− 0.14 (0.17)	1 month	23.27 (1.16)	23.14 (1.15)	< 0.05
Lopez et al.^27^	No glaucoma	− 0.19 (0.05)	1 month	﻿25.10 (3.19)	﻿24.88 (3.16)	> 0.05
Bernardo et al.^28^	No glaucoma	− 0.01 (0.08)	2 months	23.69 (1.31)	23.69 (1.31)	> 0.05
Chang et al.^26^	No glaucoma	− 0.10 (0.15)	3 months	24.22 (1.59)	24.13 (1.93)	< 0.05

In our study we also found different AL change in control and OAG groups postoperatively, and this could be related to different scleral rigidity in glaucomatous eyes. Experimental animal studies, as presented by Oglesby et al^41^, show that increased IOP and glaucoma cause fibroblast proliferation in mice sclera. Coudrillier et al. found, that human glaucomatous eyes had higher scleral fiber stiffness comparing to non-glaucomatous eyes^42^. Kim et al., found in their study, that glaucomatous eyes had larger and more deeply curved posterior poles of an eye, indicating posterior scleral rearrangement^43^. This could be possibly due to elastine alteration in posterior pole, which appears under IOP stress conditions in glaucoma patients^44, 45^.

Another important factor to consider is that changes in choroid could also alter AL in eyes after cataract surgery. Yilmaz et al. found in their study that choroid slightly increases in thickness and does not return to its’ previous value^46^. Chen et al. also found increased choroidal vascularity after phacoemulsification^47^, observing AL decrease after phacoemulsification in the same patients^47^. More research is needed to fully elucidate the choroid and its influence on AL in cataract patients.

One of the advantages of our study was that we compared both IOP change and ACA measurements between cataract patients with and without OAG both pre- and postoperatively. Comparatively, most studies available in the literature evaluated only OAG without a control group, and/or OAG was compared to an angle closure glaucoma cohort. In our study we also performed precise biometrical evaluation pre- and postoperatively in comparison with ACA change. Our study is not without limitations however, as we did not include trabecular meshwork or choroidal thickness assessments. Additionally, a longer follow up duration may provide more precise information about the stability of observed changes and their influence on risk for OAG progression.

## Conclusions

In our study IOP change after cataract surgery was more pronounced in OAG cataract patients than in non-OAG cataract patients. The OAG patients were more likely to have IOP change ≤ − 3.0 mmHg than the patients without OAG. Interestingly, the IOP reduction did not correlate with preoperative ACA width in either group. The OAG group had a tendency of narrower ACA before cataract surgery, but overall anterior chamber parameters were similar among cataract and cataract with open angle glaucoma patients pre- and postoperatively. The ACD change had a moderative positive connection with ACA parameters in control group, but this was not observed in OAG group. AL was shorter postoperatively in control group, but remained similar in the OAG group. Non-glaucomatous cataract patients and OAG cataract patients had similar biometrical and ACA characteristics preoperatively, but the postoperative structural dynamics differed, suggesting different postoperative ocular tissue adaptation. Our analysis therefore suggests IOP reduction is greater post cataract removal is in OAG patients, but larger longitudinal studies are needed to understand the duration of IOP reduction. Further, we suggest designing a study to include trabecular meshwork and posterior choroid evaluations to fully elucidate cataract removal and its impact on IOP, ocular structure, and OAG progression.

## Methods

The prospective observational case-control study was conducted in Lithuanian University of Health Sciences, Kaunas, Lithuania. The Kaunas Regional Biomedical Ethics Committee approved all study procedures (No. BE-2-52) and participants signed an informed consent. The study protocol adhered to the tenets of Declaration of Helsinki.

Our study included 40 patients: the control group consisted of 25 patients and the OAG group consisted of 15 patients.

To detect the difference of 0.3 (SD = 0.2) mm change in ACA opening distance (AOD500), we needed at least 11 participants in each group (α = 0.05, power 90%)^13^. To detect an IOP difference of 2.3 mmHg (SD 2.0), we needed at least 12 participants in each group (α = 0.05, power 80%)^48^.

Inclusion criteria for all subjects included: age > 40 years, IOP < 21 mmHg, vision ≥ 0.2 decimal, diagnosed with cataract, scheduled for cataract surgery, open ACA gonioscopically (Schaffer III-IV), OAG group with antihypertensive glaucoma medical treatment for more than 2 years. The control group consisted of cataract patients without other ophthalmological pathology and the (OAG) group consisted of cataract patients who were previously diagnosed with OAG for more than 2 years and were treated medically with controlled IOP. Exclusion criteria included previous ocular surgery or laser treatment; systemic conditions, such as diabetes mellitus, uncontrolled arterial hypertension; other ocular conditions, such as closed angle glaucoma, age related macular degeneration, diabetic retinopathy, mature cataract (we needed to be able to use IOLMaster for biometry), lens subluxation or swelling (to avoid lens induced glaucoma), high myopia, previous inflammatory eye diseases, ACA pathology.

All subjects had demographical data evaluated: age, gender and ethnicity data collected, underwent full ophthalmic examination, Goldmann applanation tonometry for IOP, ocular biometry (IOLMaster 700 v1.7), using “phakic” option preoperatively and “pseudophakic” option postoperatively. We also performed AS-OCT (DRI OCT Triton plus (Ver.10.13). AS-OCT was performed in semi-dark conditions without pupil dilation. The ACA was scanned at 0° and at 180° three times each side. We performed scans of 90° and 270°, but we did not include them into our study due to scleral artefacts. Ocular biometry measurements were performed without pupil dilation in a well-lit room. Ocular tonometry was performed as the last test to avoid artefacts in AS-OCT and biometry. The measurements were obtained before and repeated 6 months after phacoemulsification and IOL implantation.

All of the participants underwent uneventful phacoemulsification and IOL implantation. All of the surgeries were performed by one surgeon (L.K.) with temporal clear corneal incision (2.4 mm). The surgery followed viscoelastic material insertion, continuous curvilinear capsulorhexis (approximately 5.5 mm diameter), hydrodissection, phacoemulsification of the nucleus and aspiration of cortex. The IOL was inserted into the capsular bag. We used Tecnis® Monofocal 1-Piece Model ZCB00 lenses (Johnson and Johnson Vision, United States of America), Alcon AcrySof® IQ lenses (Alcon, Switzerland and United States of America) and EnVista® lenses (Bausch + Lomb, United States of America).

The ΔIOP was calculated postoperative IOP minus preoperative IOP (mmHg).

The AS-OCT measurements were performed using Fiji program package^49^. All of the measurements were performed by one grader (G.P.). The intra-observer repeatability of randomly chosen 30 images was excellent (PCC = 0.9).

**Figure 4:**
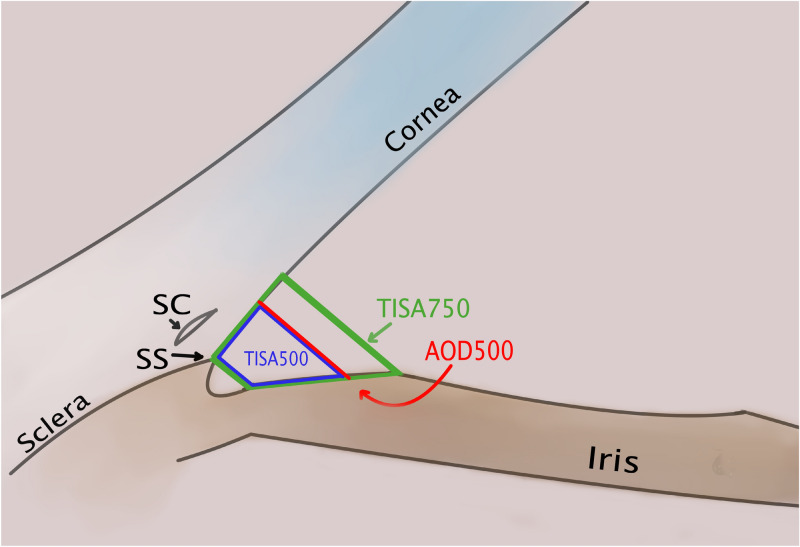
Measurements of anterior angle structures: AOD500, TISA500, TISA750. *SS* scleral spur, *SC* Schlemm’s canal. (Illustration made using Clip Studio Paint PRO Ver. 1.9.10 ©CELSYS Inc. https://www.clipstudio.net/en/).

To characterize anterior chamber parameters, we used measurements, as described by Pavlin et al. (AOD)^50^, and Radhakrishnan et al. (TISA)^33^ (Fig. 4).

AOD500—angle opening distance at 500 μm from scleral spur. It is a line, beginning at a dot at 500 μm from scleral spur on corneal endothelium, perpendicular to corneal endothelium, ending on iris surface.

TISA500—trabecular-iris space area. Circumscribed area, where anterior wall is AOD500, posterior wall starts from scleral spur and is parallel with AOD500, superior wall is corneoscleral surface and inferior wall is iris surface. TISA750 is has similar boundaries with AOD500 replacement by distance 750 μm from scleral spur.

AOD500 change was measured postoperative AOD500 minus preoperative AOD500 (mm).

We included ocular biometry parameters: AL—axial length (mm), CCT—central corneal thickness (μm), ACD—anterior chamber depth (mm), lens thickness (mm) and IOL thickness (mm). ACD change was measured postoperative ACD minus preoperative ACD (mm).

Statistical analysis was performed using IBM SPSS Statistics for Windows, Version 23.0. (Armonk, NY: IBM Corp) program package. Kolmogorov–Smirnov test was used to evaluate the normality of sample distribution. Mann–Whitney U test was used for 2 non-parametric independent samples. Quantitative data was presented as Mean (SD). Spearman’s Correlation Coefficient was used for non -parametric correlations. A *p* value < 0.05 was statistically significant.

## Data Availability

All of the data is available from the corresponding author upon reasonable request.
